# Phosphorus leaching and runoff risks from non‐calcareous sandy soils with a low sorption capacity and high hydrological connectivity

**DOI:** 10.1002/jeq2.70180

**Published:** 2026-04-15

**Authors:** Maarten van Doorn, Wim de Vries, Debby van Rotterdam, Gerard H. Ros

**Affiliations:** ^1^ Nutriënten Management Instituut Wageningen The Netherlands; ^2^ Earth Systems and Global Change Group Wageningen University Wageningen The Netherlands

## Abstract

Sustainable phosphorus (P) management includes producing food within environmental boundaries for water quality. In regions where environmental boundaries are crossed, it is beneficial to identify P loss hotspots and implement mitigation measures. In this study, we assessed the risk of P losses to shallow groundwater and surface water from agricultural fields on non‐calcareous sandy soils with an exceptionally low P sorption capacity and high hydrological connectivity due to shallow groundwater levels and the presence of open trenches. Specifically, we investigated P quantity–intensity relationships in soils from two agricultural fields and monitored groundwater levels and P concentrations in both groundwater and water fluxes from open trenches. The results showed that non‐calcareous soils with low sorption capacities reach high P saturation degrees when fertilized to an agronomic optimum based on a P quantity measure. This leads to high reactive P concentrations in soil solution that can be transported to surface water via interflow, overland flow, and land drainage. In these situations, open trenches are a significant P loss pathway because they directly connect the P‐saturated topsoil to surface water, leading to P losses ranging from 1.3 to 7.5 kg P ha^−1^ year^−1^. Effective mitigation measures include reducing dissolved P losses by reducing the soil P status of fields to environmental soil P intensity thresholds through negative P balances and reducing particulate P losses by implementing erosion control measures. However, because inlet water substantially contributes to the total water discharge, within‐catchment mitigation measures may need to be complemented by upstream mitigation measures.

## Core Ideas

- Soils with a low phosphorus (P) sorption capacity are hotspots for P losses to surface water.
- Open trenches are significant P loss pathways due to strong connectivity between topsoil and surface water.
- Applying fertilizer according to recommendations based on environmental P intensity thresholds reduces dissolved P losses.
- Field and trench management reduces erosion and associated particulate P losses.

## Plain Language Summary

Phosphorus (P) is an essential nutrient for crop production, and therefore applied by farmers to soils in the form of P fertilizers and animal manure. However, P losses from agricultural fields to surface water can harm aquatic ecosystems. In this study, we investigated P losses on agricultural fields that are particularly vulnerable to P losses because of a low P binding capacity of the soil. These fields are also highly connected to surface water due to shallow groundwater levels and the presence of open trenches. We found that these soils easily saturate with P, leading to substantial losses. Open trenches are a major pathway for P to enter surface water, as they connect the P‐saturated topsoil directly to surface water. To reduce these losses, we suggest decreasing the P levels in soils to environmental thresholds, and using better field and trench management to decrease erosion.

**Abbreviations**
CSA: critical source area
DRP: dissolved reactive P
DUP: dissolved unreactive P
GWT: groundwater table
ICP‐MS: inductively coupled plasma mass spectrometry
OCV: Opsterlandse Compagnonsvaart
PSC: P sorption capacity
PSD: P saturation degree
SCV: Schoterlandse Compagnonsvaart
SOM: soil organic matter
TDP: total dissolved P
TP: total P
WFD: water framework directive

## 1 INTRODUCTION

Sustainable phosphorus (P) management requires balancing crop production with water quality objectives. From an agronomic perspective, fertilizer recommendations aim to increase the crop‐available soil P content to a target level for crop yield (Jordan‐Meille et al., 2012). In many regions of the world, particularly in western Europe, the United States, and China, a long history of fertilizing P above the crop P demand has led to the buildup of considerable soil P reserves and to increased crop yields (Sattari et al., 2012). However, it has also led to substantial P losses from agricultural fields to surface waters, contributing to eutrophication (Smith & Schindler, 2009). This has occurred to such an extent that we have crossed the planetary P boundary (Richardson et al., 2023).

In the European Union, P losses from agricultural fields to surface waters are mitigated by complying with the Water Framework Directive (WFD). The WFD aims to achieve a good chemical and ecological status for surface water bodies, with target deadlines initially set for 2015 (European Union, 2000). In the Netherlands, this original deadline has been extended to 2027, the latest possible date under the Directive, due to technical constraints in achieving the targets sooner. In the Netherlands, elevated total P concentrations in ditches, rivers, and lakes hinder the achievement of a good ecological status for roughly half of the WFD‐designated water bodies (Waterkwaliteitsportaal, 2025).

To decrease P losses from agricultural fields to surface waters, there is a need to identify P‐loss hotspots and implement the locally most effective mitigation measures. Targeting critical source areas (CSAs) is proposed as a cost‐effective method. CSAs are defined as “small areas of a field, farm, or catchment that account for most contaminant loss [to surface water] caused by these areas having a high contaminant availability and transport potential” (McDowell et al., 2025). At the catchment level, identifying CSAs requires identifying agricultural fields with high P availability and high hydrological connectivity to surface waters. Specifically, soils with a low P sorption capacity (PSC) and high hydrological connectivity (e.g. shallow groundwater levels) are identified as high‐risk soils as they easily saturate with P, leading to a high dissolved reactive P concentration in soil solution which can be transported to surface water via interflow, overland flow, and land drainage (Hall et al., 2023; McDowell & Monaghan, 2015; van Doorn et al., 2025).

To facilitate the identification of CSAs for P at different spatial scales, we recently mapped soil contents of amorphous Fe‐ and Al‐(hydr)oxides—the key soil properties determining the PSC in non‐calcareous soils—at high (25 m) spatial resolution for the Netherlands (van Doorn et al., 2024). These maps allow for the identification of soils with a low PSC that tend to reach high degrees of P saturation when used for agriculture, corresponding with an elevated risk of dissolved P losses to surface waters. Investigating P losses from these soils to surface waters is essential to identify effective mitigation measures that contribute to achieving water quality objectives.

In this study, we investigate the risk of P losses to groundwater and surface water from agricultural fields on non‐calcareous sandy soils with an exceptionally low PSC and a high hydrological connectivity due to shallow groundwater levels and the presence of open trenches. The fields are located in the catchments of two WFD‐designated surface water bodies in the Netherlands where P loading hinders achieving a good ecological quality. First, we assessed the risk of dissolved P losses to surface water through interflow, overland flow, and land drainage by investigating P quantity–intensity relationships in soils from two agricultural fields across the soil depth profile. Second, we evaluated the risk of P leaching to groundwater by monitoring groundwater levels and P concentrations. This provides insight into the potential for P transport to surface waters via matrix flow to groundwater and subsequent lateral flow to the ditch. Third, we assessed the risk of P losses via land drainage by monitoring P concentrations and water fluxes from open trenches in these fields. As we observed that open trenches were a major pathway for P losses to surface water, we assessed the potential impact of source‐ and transport‐oriented mitigation measures aiming to reduce these P losses. Specifically, we investigated the impact of reducing the soil P status to environmental soil P intensity thresholds through negative P balances (source measure) and implementing erosion control measures preventing particulate P losses (transport measure). While this study focuses on agricultural systems in the Netherlands, the mechanisms driving P losses from soils with a low P sorption capacity and strong hydrological connectivity extend well beyond the study area (Kleinman, 2017; Nair et al., 2004). As such, the insights gained into P loss pathways and the effectiveness of mitigation measures are broadly applicable.

## 2 MATERIALS AND METHODS

### 2.1 Study area

**FIGURE 1:**
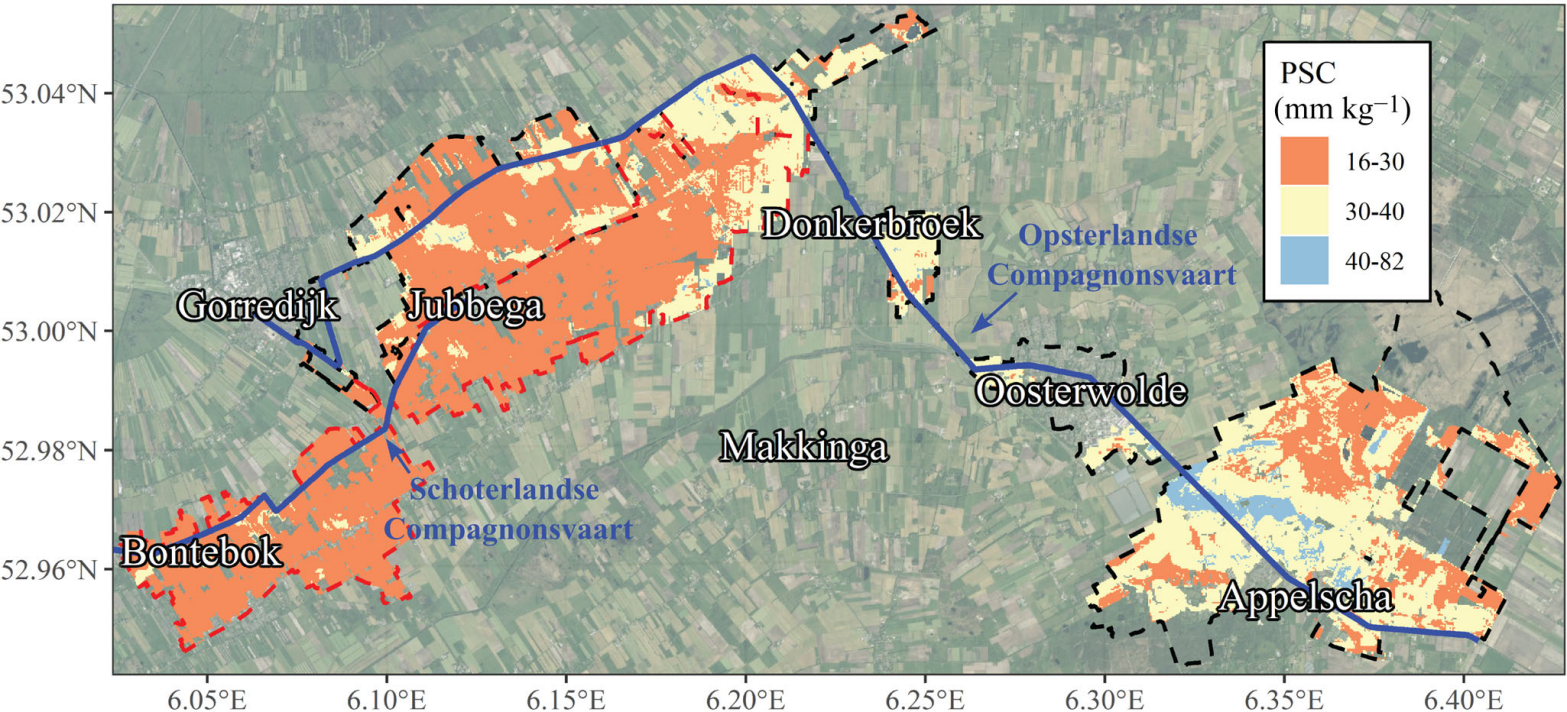
Spatial variation in the maximum P sorption capacity (PSC) of the topsoil (0–0.1 m) of agricultural fields located in the catchments of the two studied WFD‐designated surface water bodies (blue solid lines) Opsterlandse Compagnonsvaart (OCV) and Schoterlandse Compagnonsvaart (SCV). The black and red dashed lines correspond to the catchments of the OCV and SCV, respectively.

The study area is the catchments of two WFD‐designated surface water bodies in the north of the Netherlands where P loading is hindering the achievement of good ecological quality. The designated surface water bodies are the Schoterlandse Compagnonsvaart (SCV) and Opsterlandse Compagnonsvaart (OCV) in the province of Fryslân (Figure 1). Agricultural fields in the catchments are characterized by non‐calcareous sandy soils with low PSC, except 15% of the agricultural land area that is situated on peaty soils (soil map included in Figure S1). The predicted PSC for the upper (0.1 m) soil layer (van Doorn et al., 2024) ranges from 16 to 82 mmol kg^−1^, with the PSC of most fields being below 40 mmol kg^−1^ (Figure 1). Soils with a PSC below 30 mmol kg^−1^ tend to reach high P saturation degrees (PSDs) when fertilized to an agronomically optimal soil P level (van Doorn et al., 2023). The PSD expresses the extent to which the PSC is saturated with P, that is, the P loading of amorphous Fe‐ and Al‐(hydr)oxides. The needed soil P quantity level for optimum crop productivity, as determined by soil tests, is generally set equal for all soils, but in soils with a very low PSC, this P quantity level may exceed acceptable PSD levels. In the Netherlands, a P quantity content of 80–150 mg kg^−1^ in the topsoil (measured as P_AL_ in the 0‐ to 0.1‐m soil layer) has been recommended for grassland (Hoeks et al., 2005), while a P_AL_ content of 150–200 mg kg^−1^ in the 0‐ to 0.25‐m soil layer is recommended for arable land (Commissie Bemesting Akkerbouw/Vollegrondsgroenteteelt [CBAV], 2024). A PSC below 30 mmol kg^−1^ occurs only in roughly 16% of the agricultural soils in the Netherlands (van Doorn et al., 2024). In contrast, in the study area (the SCV and OCV catchments), soils with a PSC below 30 mmol kg^−1^ cover approximately 60% of the total agricultural land area (Figure 1). Farm types in the catchments mainly consist of dairy farms; roughly 90% of the agricultural area is grassland and maize (Figure S2). Circa 3150 and 2300 hectares of agricultural land are located in the catchments of the OCV and SCV, respectively (Figure 1).

**Core Ideas**

- Soils with a low phosphorus (P) sorption capacity are hotspots for P losses to surface water.
- Open trenches are significant P loss pathways due to strong connectivity between topsoil and surface water.
- Applying fertilizer according to recommendations based on environmental P intensity thresholds reduces dissolved P losses.
- Field and trench management reduces erosion and associated particulate P losses.

### 2.2 Data collection

We selected two representative agricultural fields in the western part of the study area where the estimated PSC was below 30 mmol kg^−1^, and the hydrological connectivity was high due to high groundwater levels and the presence of open trenches (trenches shown in Figure S3). On these fields, we investigated the availability of P across the soil depth profile, monitored groundwater levels, P concentrations in groundwater, and assessed water fluxes and P concentrations in water flowing from open trenches.

Soil samples were collected in the spring (May 2023) at four depths per field, with soil sampling depths ranging from the soil surface to the mean lowest groundwater table. The topsoil (0–0.1 m) was taken as a composite sample representing the entire field using a grass plot sampler, consisting of approximately 30 cores per hectare. Deeper soil layers were taken at a single representative location within the field as a point sample, consisting of a composite of circa six cores taken within a 10‐m radius around the sampling location, using an Edelman auger. The fields were not fertilized with P for at least 5 weeks before the time of sampling, to avoid fertilization strongly influencing measurements of directly available soil P. Groundwater levels and P concentrations in groundwater were measured monthly from March 2023 to February 2025 in fixed monitoring wells (filter depth 2.0–3.0 m below soil surface; see Figure S4). Groundwater samples were divided into filtered (0.45 µm) and unfiltered acidified subsamples (NEN‐EN‐ISO 5667‐3, 2024). Water fluxes of open trenches were continuously monitored from December 2023 to February 2025 using a solar‐powered water flow meter (Figure S5). Water samples were taken three to five times throughout the study, with sampling times selected to capture the observed variation in flow rates. Trench water samples were taken as unfiltered acidified samples (NEN‐EN‐ISO 5667‐3, 2024). The lower sampling frequency for trench water was mainly due to practical considerations. These samples were collected by the farmers themselves, and the sampling schedule therefore had to remain compatible with their regular farm activities.

In addition to the two intensively monitored fields, we investigated the spatial variability of soil P availability across the agricultural fields in the catchments. We took topsoil (composite) samples from 40 randomly selected fields across the catchments, comprising 16 arable and 24 grassland fields. The soil layers 0–0.1 m and 0.1–0.25 m were sampled on permanent grasslands, and the soil layers 0–0.25 m and 0.25–0.40 m were sampled on temporary grasslands and arable land.

### 2.3 Chemical analyses

Soil samples were analyzed for soil organic matter (SOM); pH_CaCl2_; directly available P (P_CaCl2_); moderately strong, reversibly bound P (P_AL_); amorphous Fe‐ and Al‐(hydr)oxides (Fe_OX_, Al_OX_); and total reversibly bound P (P_OX_). The SOM content was estimated from total organic carbon measured with dry combustion, assuming that 58% of SOM consists of total organic carbon. Before dry combustion, carbonates were removed by treating the soil sample with HCl. Carbon dioxide released by dry combustion was measured by an elemental analyzer. Both pH_CaCl2_ and P_CaCl2_ were measured by extracting the soil with a 0.01 M CaCl_2_ solution at a soil solution ratio of 1:10 (w/v) (Houba et al., 2000). Samples were shaken for 120 min at 250 rpm, after which the pH in the suspension was measured using a glass electrode. Afterward, the suspension was filtered through a 0.22 µm membrane filter. The inorganic P content of the filtrate was measured colorimetrically using a discrete analyzer (P_CaCl2,DA_), being a proxy for dissolved reactive P, and the total P content of the filtrate was measured using Inductively Coupled Plasma Mass Spectrometry (ICP‐MS; P_CaCl2,ICP_), being a proxy for total dissolved P. Note that dissolved P is commonly defined operationally as P passing a 0.45 µm filter, whereas a 0.22 µm filter was used during the CaCl_2_ extraction procedure. However, the difference in measured soluble P concentrations between the use of 0.22 µm and 0.45 µm filters has been observed to be negligible (McDowell & Sharpley, 2001). P_AL_ was measured by extraction with a 0.1 ‐ = M ammonium lactate and 0.4 M acetic acid solution (pH 3.75) at a soil solution ratio of 1:20 (w/v) (Egnér et al., 1960). Samples were shaken for 4 h at 180 rpm. The P content of the filtrate was measured colorimetrically using a discrete analyzer. Oxalate‐extractable Fe, Al, and P were measured by extraction with 0.2 M acid ammonium oxalate (pH 3.0) at a soil solution ratio of 1:20 (w/v) (Schwertmann, 1964). Samples were shaken for 2 h at 280 rpm. Total Fe, Al, and P contents of the filtrate were measured using ICP‐MS.

Groundwater samples were analyzed for dissolved reactive P (DRP, commonly called ortho‐P) and total P (TP). Trench water samples were analyzed for only TP. DRP was measured colorimetrically as the P concentration of the filtered (0.45 µm) water sample, using a discrete analyzer (NEN‐EN‐ISO 15923‐1, 2024). TP was measured as the total P concentration of the unfiltered acidified water sample, thus including both dissolved and particulate P, using continuous flow analyses and an auto‐analyzer (NEN‐EN‐ISO 15681–2, 2018). Trench water was analyzed for TP only to keep the sampling manageable for farmers, as additional analysis for DRP would have required collecting and filtering an additional sample.

### 2.4 Data evaluation

Soil contents of P_OX_ and Fe_OX_ and Al_OX_ were used to estimate both the PSC (Equation 1) and the PSD (Equation 2).

(1) $$\mathrm{PSC}\;=\alpha_{\max}\;\times \left(Fe_{\mathrm{OX}}+\;Al_{\mathrm{OX}}\right),$$

(2) $$\mathrm{PSD}\;=\frac{P_{\mathrm{OX}}}{\mathrm{PSC}}\;\times \;100,$$

where PSC refers to the P sorption capacity (mmol kg^−1^); PSD to the P saturation degree (%); Fe_OX_, Al_OX_, and P_OX_ to oxalate‐extractable Fe, Al, and P (mmol kg^−1^); and *α*_max_ to the maximal saturation factor (−). We assumed a maximal saturation factor *α*_max_ of 0.5 (Schoumans & Chardon, 2015).

The P flux leaving the fields from open trenches was estimated by multiplying the water fluxes from those trenches by the measured TP concentrations. We used the mean TP concentration per field, based on the results from water samples that were taken three to five times during the year, as we observed that the temporal variation in P concentrations was relatively low within fields but substantially differed between the two fields. Water fluxes were converted from L day^−1^ to L m^−2^ day^−1^ by assuming that the whole field was in connection with the open trenches, thus dividing the water flux by the surface area of the field. Although Field 1 is connected to a neighboring field at a slightly higher elevation, we assumed that this field did not contribute to the trench discharge, as the trench is located entirely within Field 1. For 20% of measurement days, the water flux through open trenches to surface water surpassed the daily precipitation multiplied by the surface area of the field, where precipitation data were retrieved from the nearest meteorological station “Gorredijk” (Koninklijk Nederlands Meteorologisch Instituut, 2025), located approximately 4.5 km from the two monitoring fields and at a comparable elevation (difference < 2 m). These instances were considered outliers, likely resulting from overestimated water fluxes by the flow meter. Therefore, for these days, the water flux was set equal to the precipitation recorded that day, based on the assumption that trench discharge was primarily driven by rainfall‐induced overland flow and shallow interflow.

### 2.5 Catchment scale estimation of phosphorus losses from open trenches

**FIGURE 2:**
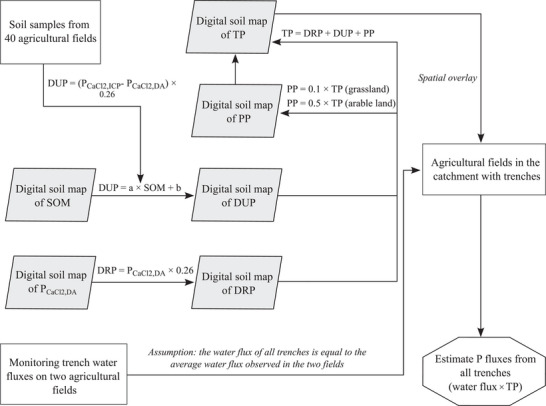
Methodological framework used to estimate P fluxes from all trenches of agricultural soils located in the Opsterlandse Compagnonsvaart (OCV) and Schoterlandse Compagnonsvaart (SCV) catchments.

Total P fluxes from open trenches of all agricultural fields in the catchments were roughly approximated in four steps (see Figure 2 for methodological framework). First, the data from the regional water authority (Wetterskip Fryslân) were used to identify which fields were connected to the surface water with open trenches. Second, the total dissolved P concentration of the trench water was estimated for each field as the sum of DRP and DUP. DRP (in mg L^−1^) was calculated by multiplying field‐specific estimates of P_CaCl2,DA_ (Verweij, 2025, in mg P kg^−1^) by a factor of 0.26. This factor is based on an empirical relationship between the soil P_CaCl2,DA_ content and the DRP concentration in soil solution in major Dutch soil types (Koopmans et al., 2006). DUP (in mg L^−1^) was estimated by (i) constructing a linear regression model predicting the difference between P_CaCl2,ICP_ and P_CACl2,DA_ from SOM, using data from the 40 sampled agricultural fields in the catchment; (ii) predicting the difference between P_CaCl2,ICP_ and P_CaCl2,DA_ for every field by applying the linear regression model on field‐specific estimates of SOM (Verweij, 2025); and (iii) converting these predictions to estimations of DUP by multiplying them by a factor 0.26, thus assuming the same empirical relationship as between P_CaCl2,DA_ and DRP (Koopmans et al., 2006). Third, TP was estimated by assuming that particulate P (PP) accounted for 10% to TP for grassland and 50% to TP for arable lands. For grassland, this assumption was based on a comparison between soil P_CaCl2,ICP_ contents and measured total P concentrations in trench water from the two monitored grassland fields, which indicated that P losses were predominantly in a dissolved form. For arable land, a PP contribution of 50% to TP was used as a rough approximation, acknowledging that PP contributions can vary substantially among fields (Hart et al., 2004). Information on whether fields consisted of arable land or grassland was obtained from the national crop parcel registration (Basisregistratie Percelen, 2025). Finally, we estimated the P flux at a daily temporal resolution by multiplying TP with the water flux (in mm and thus dependent on the area of the field), which we assumed to be equal to the average of the water flux observed in the two monitoring fields.

To identify suitable mitigation measures for reducing P losses from open trenches to surface waters, we quantified the effect of incorporating a P intensity threshold in fertilizer recommendations (source‐oriented measure), ensuring that the DRP concentration in soil solution does not exceed an environmental threshold. We defined this threshold as a P_CaCl2,DA_ content of 1–2 mg kg^−1^, corresponding to a DRP concentration of approximately 0.3–0.5 mg P L^−1^. In the Netherlands, a P_CaCl2,DA_ content of 1 mg kg^−1^ in the topsoil has been proposed as an environmental threshold for agricultural soils with high hydrological connectivity to surface waters (van Doorn et al., 2025). However, higher P_CaCl2,DA_ contents up to approximately 2 mg kg^−1^ are required to achieve agronomically desirable grass P contents (3.7 mg kg^−1^ in the first cut), according to Dutch fertilizer recommendations (van Rotterdam & Bussink, 2016). The selected P_CaCl2,DA_ threshold range of 1–2 mg kg^−1^ therefore represents a trade‐off between agronomic productivity and environmental protection. The adaptation of this P intensity threshold implies a reduction in the soil P status for soils with current P_CaCl2,DA_ contents above 1 and 2 mg kg^−1^. This can be achieved through a negative P balance (P uptake > P input).

In addition, we assessed the potential impact of erosion control measures to reduce the particulate P concentration of trench water by 50% (transport‐oriented measure). In fields with open trenches, trench design and management provide opportunities to limit sediment mobilization and associated P transport. Such measures include constructing gradual‐sloped rather than steep‐sided trenches to maintain vegetation cover within the trench (preventing bed and bank scour). In fields where particulate P predominantly enters the trenches via overland flow, maintaining adequate vegetation cover on the field and preventing soil compaction can further reduce particulate P losses.

To assess the potential contribution of agricultural mitigation measures to reduce P losses on a catchment scale and improve water quality, we evaluated the relative contribution of agricultural water fluxes to the total water fluxes to the OCV and SCV, as reported by the regional water authority (Wetterskip Fryslân, 2018, 2019). This assessment provides insight into whether incorporating a P intensity threshold in fertilizer recommendations and the implementation of erosion control measures are sufficient to substantially lower P loading to surface waters, or whether additional upstream interventions are necessary.

Estimating P losses from open trenches at the catchment scale, as well as assessing the effectiveness of mitigation measures to reduce these losses, is associated with considerable uncertainty. The upscaling approach relies on several simplifying assumptions to approximate TP concentrations and water fluxes from open trenches across all agricultural fields within the catchments. Several processes influencing P losses, such as P release kinetics and enrichment ratios, are not considered but are considered acceptable simplifications for an assessment at this spatial scale and purpose of this study. Consequently, the results should be interpreted as rough approximations intended to provide practical insight into the potential effectiveness of mitigation measures in reducing P loadings at the catchment scale.

## 3 RESULTS AND DISCUSSION

### 3.1 Soil P quantity–intensity relations are influenced by sorption capacity

The soils of the two studied fields were characterized by an exceptionally low PSC of 11–26 mmol kg^−1^ (Table 1). Amorphous Al‐(hydr)oxides were the main contributor to the PSC with Al_OX_ contents ranging from 21 to 45 mmol kg^−1^ and Fe_OX_ contents ranging from 2 to 11 mmol kg^−1^. Soil organic matter contents were relatively high for a sandy soil, circa 11% in the upper 0.1 m of the soil profile, and decreasing to 1% with increasing soil depth (1.0–2.4 m). Soil pH_CaCl2_ ranged from 4.7 to 4.9 in the topsoil, which is consistent with values typically observed in agricultural, non‐calcareous sandy soils.

**TABLE 1:** Soil organic matter (SOM), pH, oxalate‐extractable Fe, Al, P sorption capacity (PSC), P saturation degree (PSD) and soil P contents of the two investigated fields at four depths to the mean lowest groundwater table.

Depth (m)	SOM (%)	pH_CaCl2_ (–)	Fe_OX_ (mmol kg^−1^)	Al_OX_ (mmol kg^−1^)	PSC (mmol kg^−1^)	PSD (%)	P_OX_ (mg kg^−1^)	P_AL_ (mg kg^−1^)	P_CaCl2,DA_ (mg kg^−1^)	P_CaCl2,ICP_ (mg kg^−1^)
**Field 1**										
0–0.1	10.8	4.9	10	22	16	38	189	45	3.2	4.8
0.1–0.3	5.8	4.8	10	27	19	26	155	54	0.5	0.8
0.3–1	2.9	4.8	2	21	11	10	34	13	<0.1	<0.3
1–1.4	0.5	4.3	4	21	12	10	37	10	<0.1	<0.3
**Field 2**										
0–0.1	10.9	4.7	10	30	20	39	238	61	3.2	4.9
0.1–0.3	7.2	4.7	11	39	25	29	226	101	1.4	2.0
0.3–1.5	3.5	5.4	6	45	26	16	127	46	0.1	<0.3
1.7–2.3	1.3	5.4	2	20	11	11	40	18	<0.1	<0.3

Due to the low P sorption capacity (PSC, based on the low amorphous Fe‐ and Al‐(hydr)oxide content), even a low content of total P and moderately strong bound P (P quantity) resulted in a high content of directly available P (P intensity) and thus a high DRP concentration in soil solution. Specifically, in the upper 0.1 m of the soil profile, P availability was characterized by a total reversibly bound P content (P_OX_) of 189–238 mg kg^−1^, a moderately strongly bound P content (P_AL_) of 45–61 mg kg^−1^, and a directly available P content (P_CaCl2,DA_) of 3.2 mg kg^−1^. P availability decreased with increasing soil depth, reflecting the greater influence of (historic) P fertilization on the upper soil layers compared to deeper soil layers. In the deepest sampled soil layer, the PSC was exceptionally low (11–12 mmol kg^−1^). The total reversibly bound P content was, however, also exceptionally low (P_OX_ of 37–40 mg kg^−1^), leading to a low directly available soil P content (P_CaCl2,DA_ content below 0.1 mg kg^−1^).

The high SOM content in the topsoil seemed to increase the proportion of P intensity to P quantity, likely due to competition between SOM and P for sorption sites, leading to an increase in P_CaCl2,DA_. In addition, the difference between P_CaCl2,ICP_ and P_CaCl2,DA_ increased with increasing SOM contents, implying that DUP makes an increasing contribution to the total dissolved P concentration in soil solution as SOM contents increase.

**FIGURE 3:**
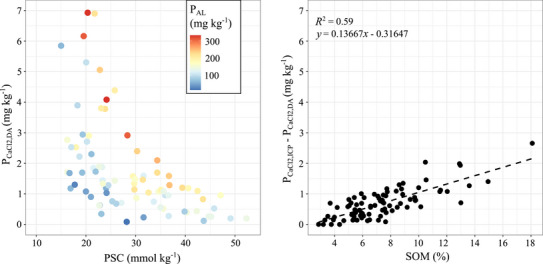
The relation between P_CaCl2,DA_ and the P sorption capacity (PSC) (left) and between the difference of P_CaCl2,ICP_ and P_CaCl2,DA_ and soil organic matter (SOM) (right), based on data from 40 agricultural fields located in the catchments. The black dashed line corresponds to a linear regression line.

The influence of the PSC on soil P quantity–intensity relations was also observed in the 40 sampled soils across the catchments. Indeed, P_CaCl2,DA_ contents increased with a decreasing PSC (Figure 3). Especially in soils with PSC values below 30 mmol kg^−1^, P_CaCl2,DA_ contents sharply increased, even at relatively low P_AL_ contents, up to a maximum of 7.0 mg kg^−1^. This implies that soils with a low PSC have relatively high risks of dissolved P losses to surface water through overland flow, interflow and land drainage, especially when (i) the hydrological connectivity between (top) soil and surface water is high, for example, due to high groundwater levels and the presence of open trenches; and (ii) available P contents are high in both the topsoil and subsoil.

Similarly to the two investigated agricultural fields (Table 1), the difference between P_CaCl2,ICP_ and P_CaCl2,DA_ (proxy for DUP) linearly increased with increasing SOM contents (*R* ^2^ = 0.59, Figure 3). The difference between P_CaCl2,ICP_ and P_CaCl2,DA_ ranged from 0 to 2.7 mg kg^−1^ with SOM contents ranging from 2.9% to 18% (Figure 3). This implies that soils with high SOM contents have a substantial potential for DUP losses, again depending on hydrological connectivity.

### 3.2 Short‐ and long‐term risks of phosphorus leaching to groundwater

**FIGURE 4:**
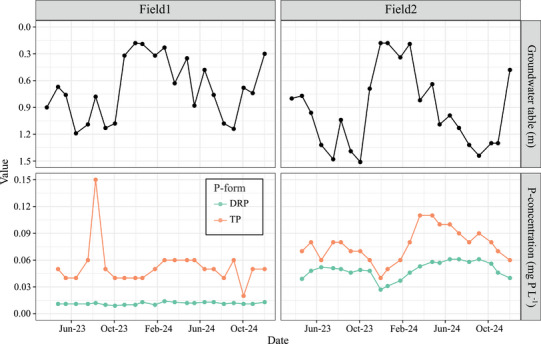
Trends in groundwater levels (top graphs) and total P (TP) and dissolved reactive P (DRP) concentrations in deep groundwater (bottom graphs) over time for the two investigated agricultural fields.

Both fields showed a high long‐term and a low short‐term risk of P leaching to groundwater via matrix flow and subsequent P transfer to surface waters via interflow. This indicates that current dominant pathways for P transfer from these fields to surface waters are overland flow, shallow interflow, and land drainage, rather than matrix flow to (deep) groundwater and subsequent P transfer to surface water via interflow. Regarding the long‐term risk of P leaching, the average PSD from the soil surface to the highest observed groundwater level (only 18 cm below the soil surface, Figure 4) exceeded the environmental threshold of 25% for Dutch non‐calcareous sandy soils, which was established in the 1990s for the protection of groundwater and surface water quality (van der Zee et al., 1990a, 1990b). Specifically, the average PSD values within this 18 cm depth were 33% for Field 1 and 35% for Field 2 (Table 1 and Figure S6). The environmental PSD threshold of 25% corresponds to a DRP concentration of 0.1 mg P L^−1^ in soil solution, assuming a conditional affinity of ortho‐P to bind to soil in the Langmuir sorption isotherm of 35 m^3^ mol^−1^ (van der Zee et al., 1990a, 1990b). Therefore, when the PSD exceeds 25% (as found for the two fields), the DRP concentration is expected to exceed 0.1 mg P L^−1^ when P is distributed evenly across the soil profile. This implies a long‐term risk of P leaching to shallow groundwater.

In contrast, current groundwater P concentrations (filter depth 2.0 to 3.0 m below the soil surface, Figure S4) suggest a low short‐term risk of P leaching to deep groundwater. Specifically, DRP concentrations ranged from 0.01 to 0.06 mg P L^−1^ and TP concentrations from 0.02 to 0.15 mg P L^−1^ (Figure 4). Those low P concentrations in deep groundwater align with the low soil P_CaCl2_ contents in the deeper soil layers. At the filter depth, soil P_CaCl2,ICP_ contents are below the detection limit (Table 1).

### 3.3 Field‐scale estimation of phosphorus losses from open trenches

**FIGURE 5:**
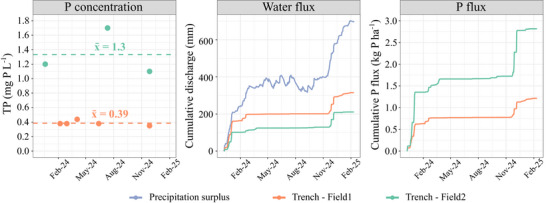
Total P concentrations of the trench water (left), water discharge of the trenches (middle), and the P flux of the trenches (right) over 1 year. For context, data on the cumulative precipitation surplus (precipitation minus evaporation) is included (middle), since this affects water discharge from agricultural fields to both groundwater and surface water.

Open trenches directly connect the P‐saturated topsoil to the ditch, leading to substantial P losses through land drainage. The TP concentrations of the trench water were high, ranging from 0.3 to 0.4 mg P L^−1^ for Field 1 and from 1.1 to 1.7 mg P L^−1^ for Field 2 (Figure 5). Overall, TP concentrations were not significantly affected by flow rate, except for a higher TP concentration in Field 2 at a low flow rate (Figure S7). For Field 1, TP concentrations were below expected based on the soil P_CaCl2,ICP_ content of the upper 0.1 m of the soil (4.8 mg kg^−1^), for which a total dissolved P (TDP, sum of DRP and DUP) concentration of roughly 1.2 mg P L^−1^ was expected (Table 1). This indicates that the trench water partially consists of interflow from the 0.1–0.3 m soil layer, where P_CaCl2,ICP_ contents are substantially lower than in the topsoil (0.5 mg kg^−1^). For Field 2, TP concentrations were roughly equal to the expected TDP concentration of circa 1.2 mg P L^−1^ based on the soil P_CaCl2,ICP_ contents of the upper 0.1 m soil layer. Notably, the decrease in P_CaCl2,ICP_ contents from the 0–0.1 m to the 0.1–0.3 m soil depth layer is less pronounced in Field 2 than in Field 1. This difference may partly explain the higher TP concentrations observed in Field 2, assuming that trench water consists of a mixture of overland flow and interflow from the 0–0.3 m soil layer.

Comparing the expected TDP concentrations derived from topsoil P_CaCl2,ICP_ contents with the measured TP concentrations suggests that a substantial fraction of TP consists of TDP (circa 70%–100%), indicating a relatively minor contribution of particulate P. This contribution of TDP to TP is higher than earlier observations on a heavy clay soil in the Netherlands (van der Salm et al., 2012). In that field trial, the contribution of DRP and DUP accounted only for 6%–13% and 9%–23% of TP, respectively. The rest of the TP was accounted for as particulate P. This can be explained by differences in soil texture, as clay particles are more susceptible to erosion than sand particles. Because erosion preferentially transports these fine, clay‐sized particles, which generally have a high P sorption capacity due to their large specific surface area, the P content of eroded sediment is typically higher than that of the bulk soil, leading to an increased contribution of particulate P to TP (Sandström et al., 2020) compared to sandy soils.

Water discharge and associated P losses from open trenches occurred predominantly during the winter period, when both the precipitation surplus and groundwater levels were high (Figure 5). For the year 2024, estimated annual P fluxes varied between 0.81 and 2.1 kg P ha^−1^ for Field 1 and Field 2, respectively (Figure 5). This is slightly lower (Field 1) or comparable (Field 2) to the range of 0.91–6.0 kg P ha^−1^ reported in the 5‐year study on grassland on a heavy clay soil (van der Salm et al., 2012).

### 3.4 Catchment‐scale estimation of phosphorus losses from open trenches

**FIGURE 6:**
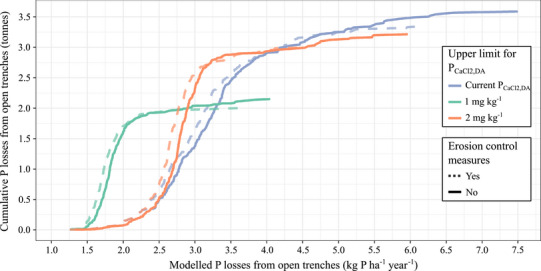
Relationship between the field‐scale P losses from open trenches and cumulative P losses from open trenches across the catchments for the year 2024 for the current situation (blue line) and when an upper environmental threshold would be used for P_CaCl2,DA_ of 1 mg kg^−1^ (green line) and 2 mg kg^−1^ (orange line). The dashed lines show the effect of erosion control measures reducing the particulate P concentration of the trenchwater by 50%.

Considering all agricultural fields with open trenches located in the catchments, the total annual P loading was estimated at 3.6 tonnes for the year 2024, with field‐specific P losses ranging from 1.3 to 7.5 kg P ha^−1^ year^−1^ (Figure 6). At the catchment level, annual P loadings from open trenches were estimated at 1.9 and 1.7 tonnes P year^−1^ for the OCV and SCV catchment, respectively.

A limited number of fields were identified as hotspots for P losses from open trenches, with losses ranging from 4.0 to 7.5 kg P ha^−1^ year^−1^ (Figure 6). On a P loss per‐hectare basis, those fields could be classified as CSAs. However, those fields only accounted for circa 20% of the total P loading from open trenches, indicating that targeting only these CSAs is insufficient to substantially decrease P loading to surface water; soils with more moderate P losses on a per‐hectare basis likely need to be targeted as well.

### 3.5 Reducing phosphorus loadings from open trenches

Possible pathways to reduce P losses from open trenches include (i) decreasing the DRP concentration of the trench water by incorporating an environmental soil P intensity threshold in fertilizer recommendations (source‐oriented measure) and (ii) decreasing the particulate P concentration of the trench water by implementing erosion control measures (transport‐oriented measure).

An environmental soil P intensity threshold could be readily incorporated in fertilizer recommendations in the Netherlands, as routine agronomic soil testing already includes the combined measurement of P quantity (P_AL_) and P intensity (P_CaCl2,DA_). These combined indicators are currently used both to determine maximum allowable P application rates for environmental purposes (Rijksdienst voor Ondernemend Nederland, 2026) and to refine agronomic fertilizer recommendations for grassland (Bussink et al., 2011) and arable land (CBAV, 2026). To avoid reaching high DRP concentrations in soil solution, an environmental P_CaCl2,DA_ threshold of 1 mg kg^−1^ for the topsoil has been proposed for soils with a high hydrological connectivity (van Doorn et al., 2025). This environmental P_CaCl2,DA_ threshold prevents soils with a low PSC from reaching high PSD values, as shown by the observed correlation between the P_CaCl2,DA_ and the PSC for the fields in the catchments (Figure 3). In this study area, reducing the soil P status of fields with P_CaCl2,DA_ contents above 1 mg kg^−1^ to the environmental threshold of 1 mg kg^−1^ through negative P balances would target most fields (covering 99% of the total agricultural land area), as P_CaCl2,DA_ contents are generally above 1 mg kg^−1^ due to the PSC of most fields being below 40 mmol kg^−1^ (Figures 1 and 3). We estimate that using a P_CaCl2,DA_ threshold of 1 mg kg^−1^ would reduce P loading from open trenches by 40%, that is, from 3.6 to 2.1 tonnes P year^−1^ (Figure 6). However, it could lead to suboptimal conditions for crop growth, particularly on soils with an exceptionally low PSC and binding affinity. In those soils, P quantity contents are low, therefore requiring higher P intensity contents for optimal crop growth (Ehlert et al., 2003). For example, in Field 1, a low P_AL_ content of 45 mg kg^−1^ was associated with a high P_CaCl2,DA_ content of 3.2 mg kg^−1^ in the upper 0.1 m soil layer (Table 1). According to Dutch fertilizer guidelines (van Rotterdam & Bussink, 2016), P_CaCl2,DA_ contents of approximately 2 mg kg^−1^ are necessary at such P quantity contents to ensure desirable grass P contents. An environmental P_CaCl2,DA_ threshold of 2 mg kg^−1^ would therefore be more agronomically feasible compared to a threshold of 1 mg kg^−1^. However, this would target a smaller proportion of fields (53% of agricultural land area), which mainly consists of soils with P_CaCl2,DA_ contents from 2 to 7 mg kg^−1^ due to a PSC below 30 mmol kg^−1^ (Figure 3). Targeting those fields only would result in a reduction of circa 10% in the total P loading from open trenches (Figure 6).

Although reducing broad‐field soil P intensity contents to environmental thresholds would be effective in decreasing DRP losses, the potential trade‐off with agricultural productivity (encompassing both yield and feed quality) suggests that alternative or complementary mitigation measures should also be explored. Such measures could complement or partly substitute a broad‐field soil P drawdown strategy. Examples include the use of iron‐filters in open trenches, selectively lowering soil P intensity contents in the trench zone (e.g. through buffer strips), establishing vegetation in trenches or along buffer strips to enhance P uptake, avoiding fertilization during periods of expected high water discharge through trenches and temporarily closing trenches following fertilization. These complementary strategies could help reduce P losses via trenches while safeguarding crop productivity.

Erosion control measures should also be considered to reduce P losses from open trenches. High P losses can occur even when DRP concentrations in soil solution remain low, especially in erosion‐prone soils where particulate P contributes significantly to the total P concentration. Assuming that particulate P makes up for 10% and 50% of the TP concentration for grasslands and arable lands, respectively, implementing erosion control measures which reduce the particulate P concentration with 50% would approximately lead to a 10% reduction in P losses from open trenches in the current situation, and a 45% reduction in P losses when combined with the implementation of the strictest environmental soil P_CaCl2,DA_ threshold of 1 mg kg^−1^ (Figure 6).

Mitigation measures aimed at reducing P losses from agriculture may need to be supplemented with upstream mitigation measures to adequately improve surface water quality of the two waterbodies. In the OCV catchment, leaching and runoff from agricultural fields accounted for approximately 30%–60% of the total water discharge, depending on the hydrological subregion, while inlet water accounted for circa 20%–70% (Figure S8). In the SCV catchment, agriculture contributed only 10%–40%, while the contribution of inlet water accounted for circa 55%–90%. This implies that P loading to these water bodies, depending on the hydrological subregion, is strongly influenced by inlet water, with the magnitude depending on the P concentration of the inlet water. Therefore, within‐catchment mitigation measures alone may be insufficient, and effective improvements in surface water quality likely require assessments and mitigation at the watershed scale rather than at individual catchments.

## 4 CONCLUSIONS

Non‐calcareous sandy soils with a low PSC and strong topsoil‐surface water connectivity are hotspots for P loss. These soils easily saturate with P, thereby increasing the risk of dissolved P losses via interflow, overland flow, and land drainage. The risk of dissolved P loss is enhanced in soils with high SOM contents due to (i) increased DRP concentrations through competition for sorption sites; and (ii) increased DUP concentrations. Open trenches, which directly connect the P‐saturated topsoil to the ditch, are a major loss pathway for dissolved and particulate P. In the study area, estimated P fluxes from open trenches correspond to 1.3–7.5 kg P ha^−1^ year^−1^. These losses can potentially be reduced up to 45% by implementing source‐ and transport‐oriented measures: decreasing dissolved P losses by reducing the soil P status of fields through negative P balances (source‐oriented) and decreasing particulate P losses by erosion control measures (transport‐oriented). Further research is recommended to assess the extent to which environmental soil P thresholds differ from agronomic optimal soil P levels on low PSC soils. To achieve significant improvements in surface water quality, mitigation measures targeting agricultural fields within the catchments may need to be complemented by upstream mitigation measures.

## AUTHOR CONTRIBUTIONS

**Maarten van Doorn**: Conceptualization; formal analysis; investigation; methodology; visualization; writing—original draft; writing—review and editing. **Wim de Vries**: Methodology; supervision; writing—review and editing. **Debby van Rotterdam**: Methodology; supervision; writing—review and editing. **Gerard H. Ros**: Conceptualization; methodology; project administration; supervision; writing—review and editing.

## CONFLICT OF INTEREST STATEMENT

The authors declare no conflicts of interest.

## Supporting information



Supporting Information

## Data Availability

Data will be made available upon request.
