# Supplementary material for: Phosphorus leaching and runoff risks from non‐calcareous sandy soils with a low sorption capacity and high hydrological connectivity
Source: J Environ Qual. 2026 Apr 15;55:e70180. doi: 10.1002/jeq2.70180 (PMC13084165; doi:10.1002/jeq2.70180)
Supplement: Supplementary file 1 — Supporting Information [file JEQ2-55-0-s001.docx]

***Supporting information of***

Phosphorus leaching and runoff risks from non-calcareous sandy soils with a low sorption capacity and high hydrological connectivity

Maarten van Doorn^a,b^*, Wim de Vries^b^, Debby van Rotterdam-Los^a^ and Gerard H. Ros^a,b^

^a^Nutriënten Management Instituut, Nieuwe Kanaal 7C, 6709PA Wageningen, the Netherlands; ^b^Earth Systems and Global Change Group, Wageningen University, P.O. Box 47, 6700AA Wageningen, the Netherlands

**Contents**

[Supplementary Figures 3](#_Toc222476330)

[References 7](#_Toc222476331)

# Supplementary Figures


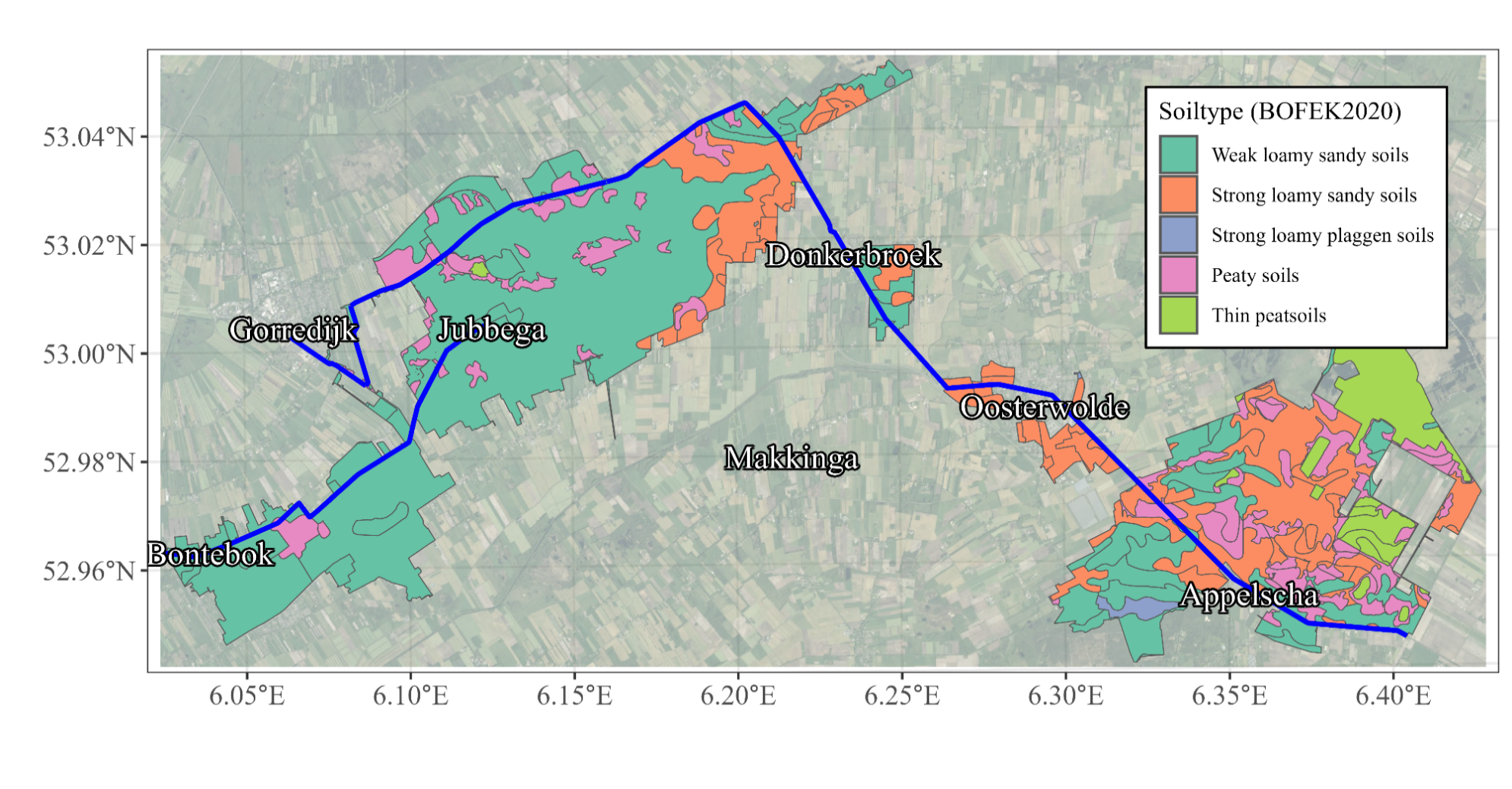


Figure S1. Soil types in the catchment of the Schoterlandse and Opsterlandse Compagnonsvaart (Heinen et al., 2022).


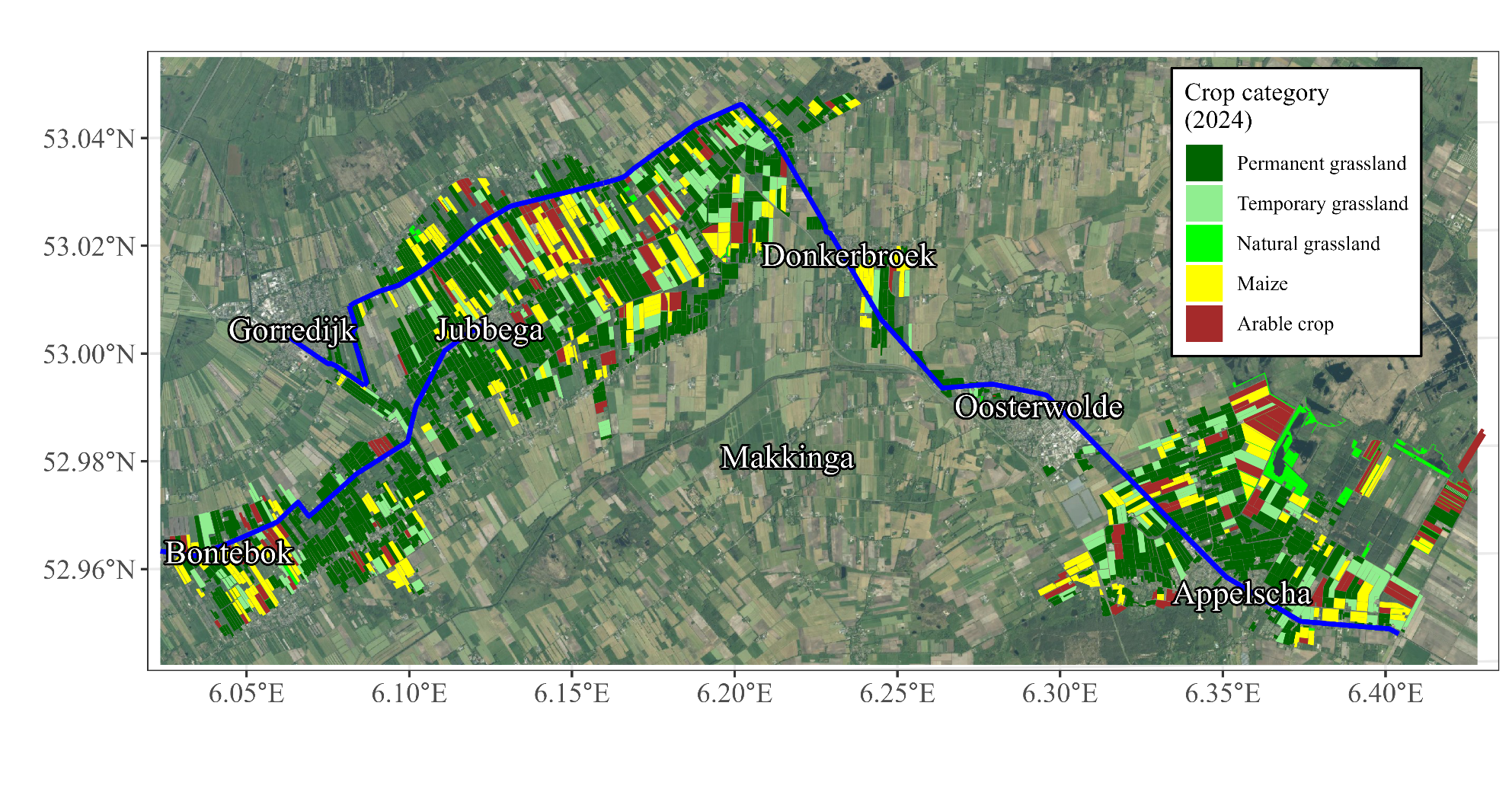


Figure S2. Main tilled crop for the year 2024 on the agricultural soils located in the catchment of the Schoterlandse and Opsterlandse Compagnonsvaart (BRP, 2025).


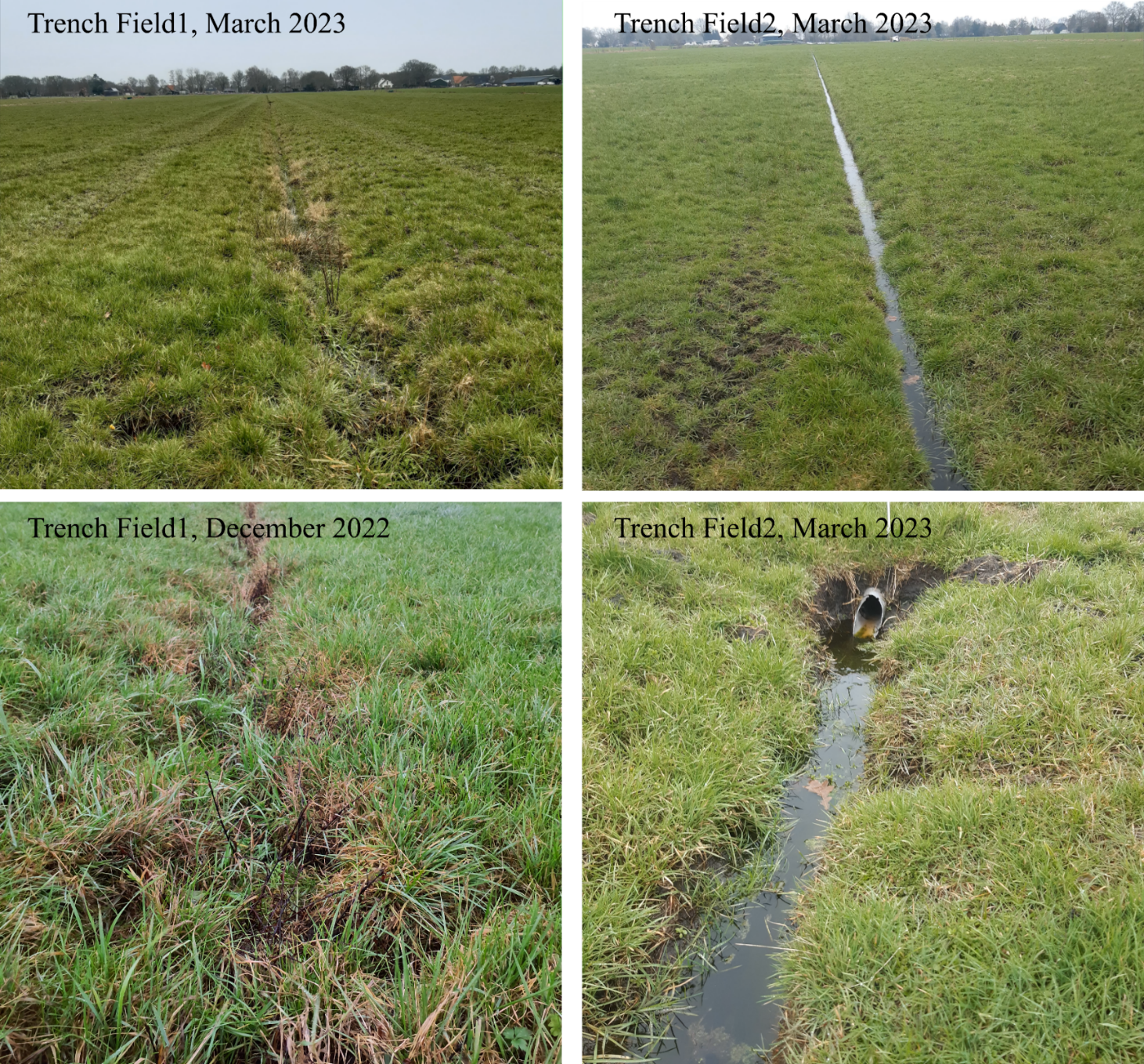


Figure S3. Open trenches of Field1 (left) and Field2 (right).


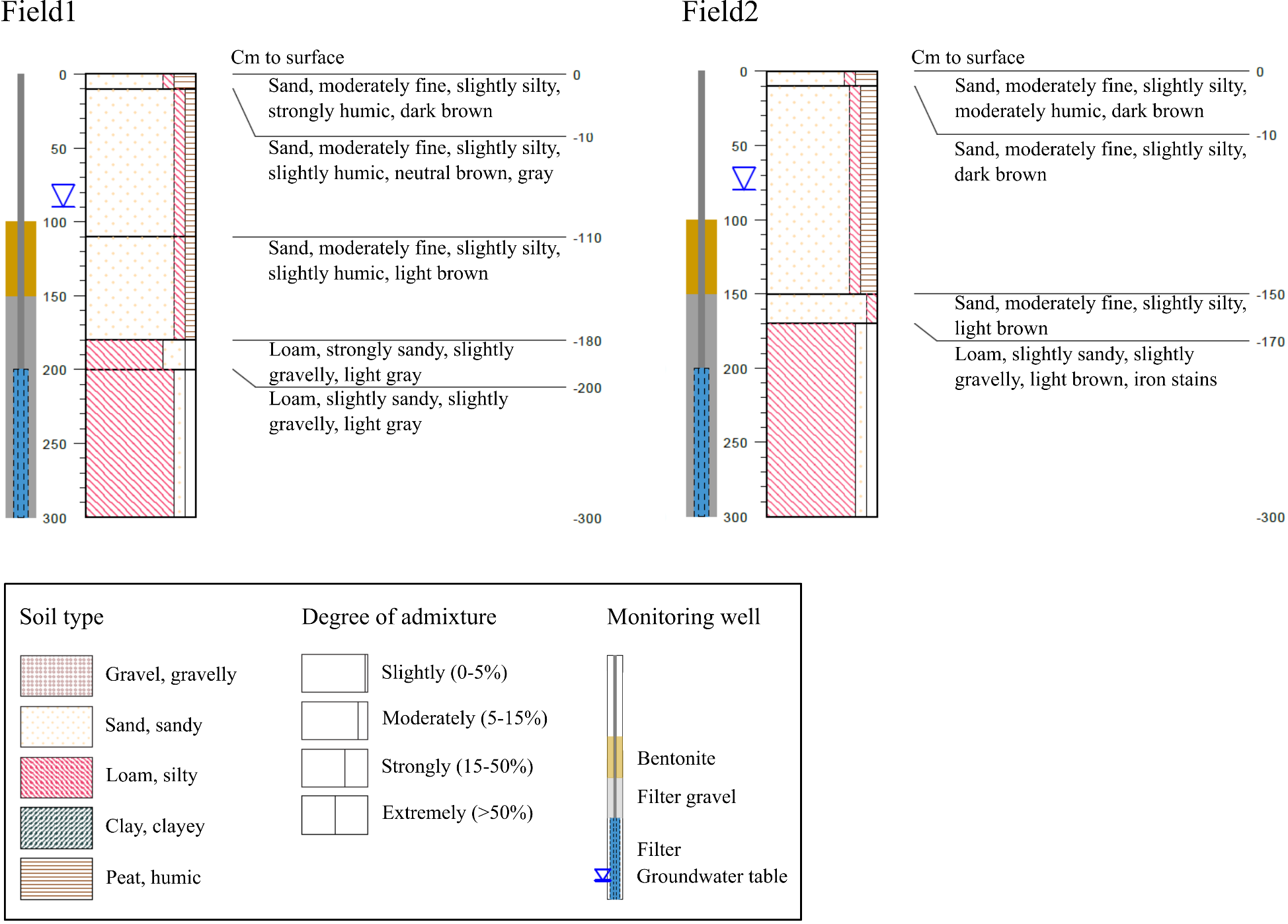


Figure S4. Description of the soil profile at the groundwater monitoring well locations.


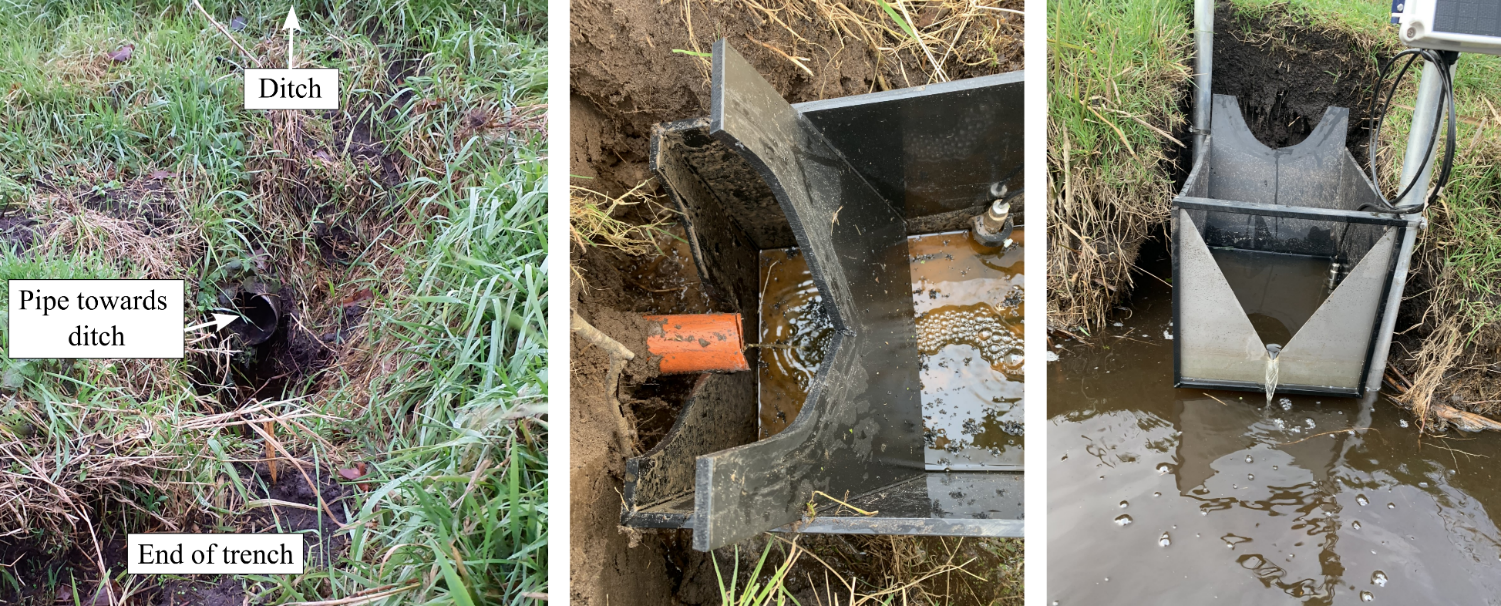


Figure S5. Solar-powered water flow meter. The open trenches were connected to the ditch via a drainage pipe running under a headland along the ditch (left), which allows farmers to drive around the trenches. The water meters were installed on the end of the drainage pipes.


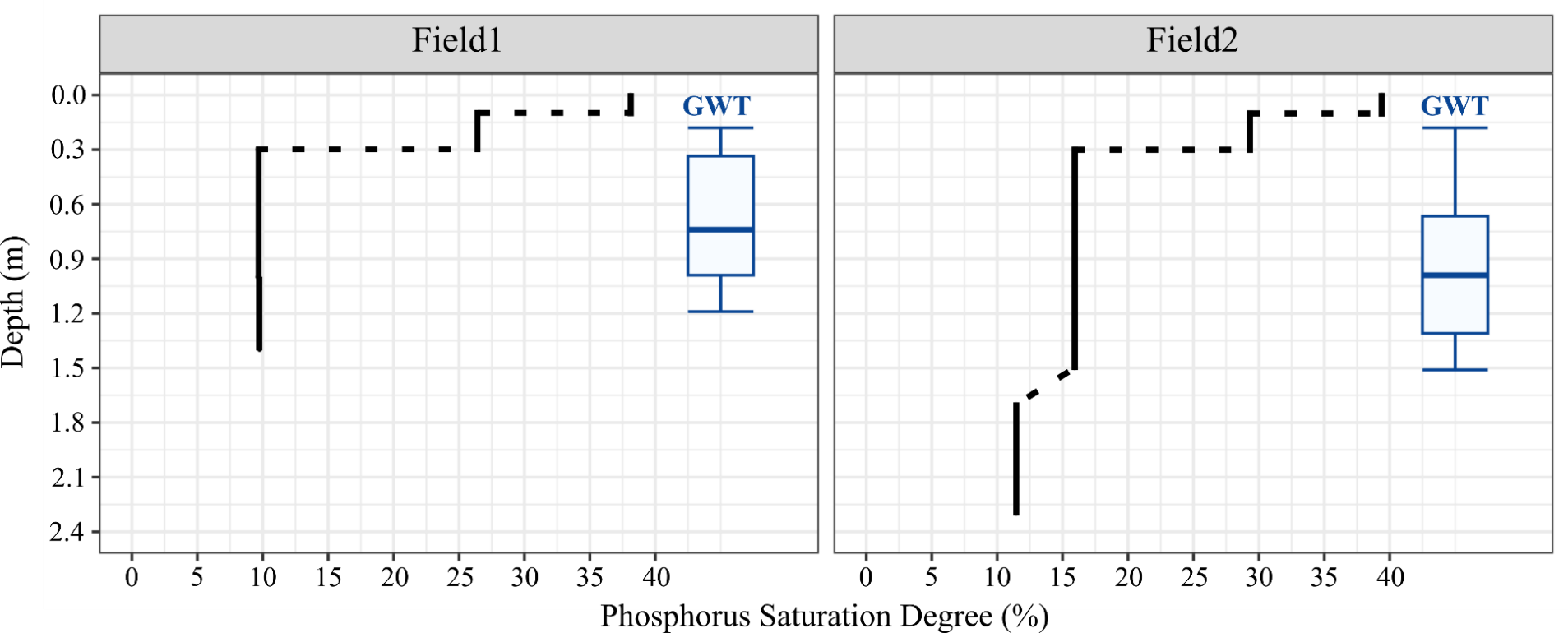


Figure S6. The change in the phosphorus saturation degree with soil depth for the two investigated agricultural fields, together with the distribution of the groundwater table (GWT).


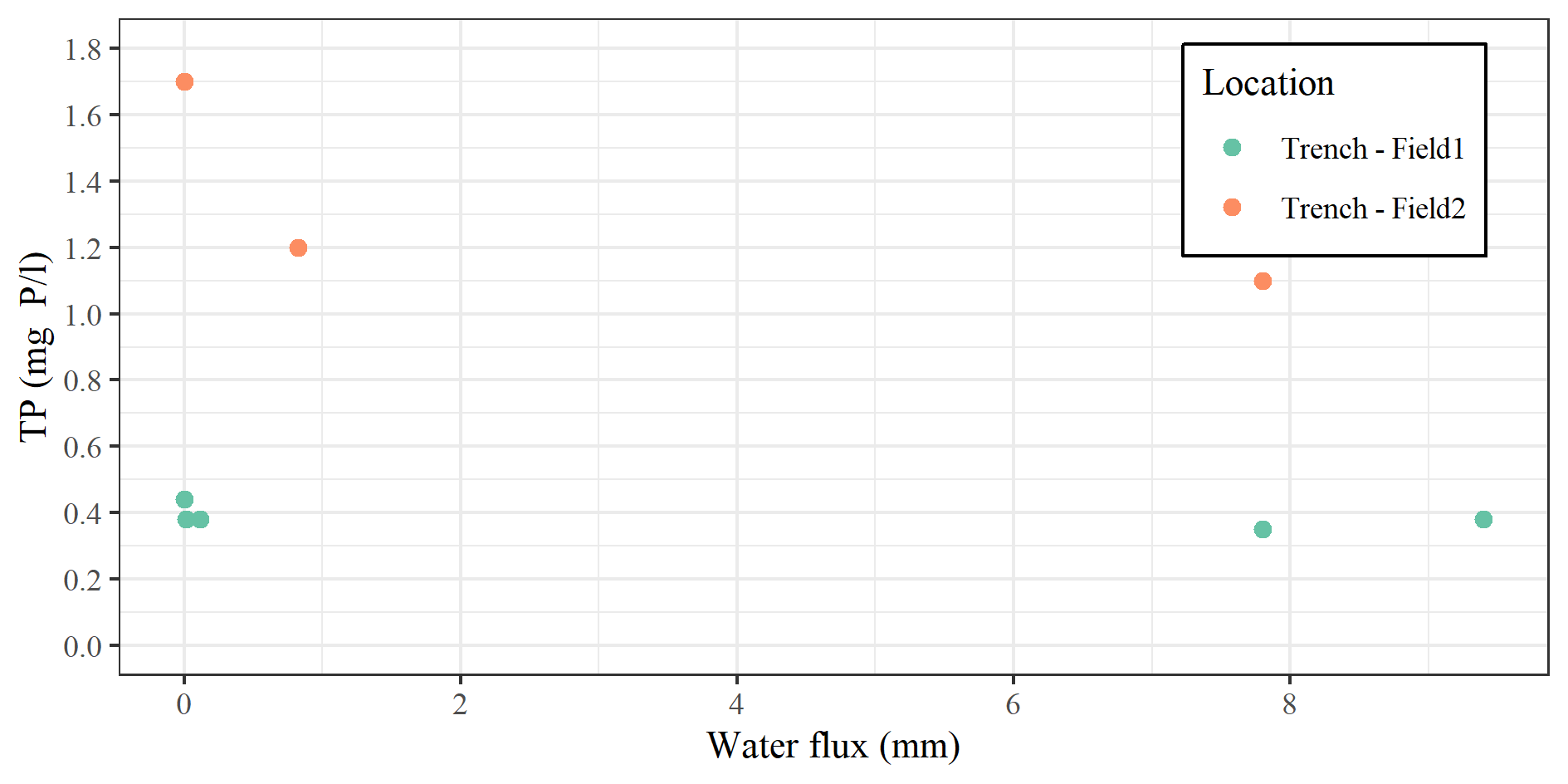


Figure S7. Daily water flux through open trenches versus total P concentrations (TP) for the two monitoring fields.


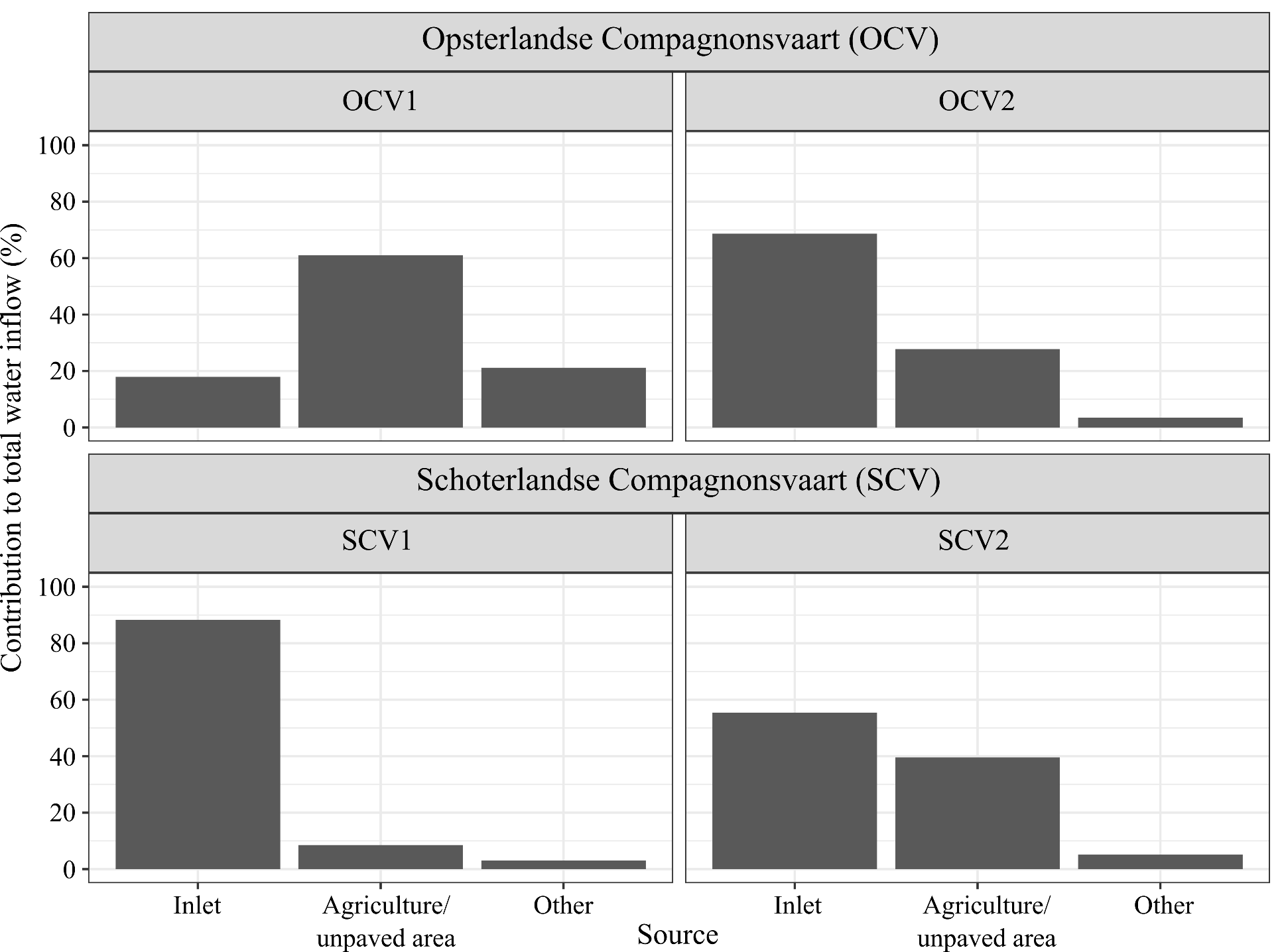


Figure S8. The contribution of inlet water, agriculture (runoff, leaching and drainage from the unpaved area) and other sources (precipitation, seepage, runoff from paved areas) to the total annual water inflows to hydrological subregions of the OCV (OCV1 and OCV2, data from 1996-2016) and SCV (SCV1 and SCV2, data from 2014-2017). The results are based on waterbalances of the regional water authority underlying the water system analyses reports (Wetterskip Fryslân, 2018, 2019). The hydrological subregions were delineated by the water authority based on, amongst others, the locations of inlets, outlets and weirs.

# References

AHN. (2025). *Actueel Hoogtebestand Nederland*. www.ahn.nl

BRP. (2025). *Basisregistratie gewaspercelen (BRP)*. Basisregistratie Gewaspercelen (BRP). https://www.pdok.nl/introductie/-/article/basisregistratie-gewaspercelen-brp-

Heinen, M., Mulder, H. M., Bakker, G., Wösten, J. H. M., Brouwer, F., Teuling, K., & Walvoort, D. J. J. (2022). The Dutch soil physical units map: BOFEK. *Geoderma*, *427*, 116123. https://doi.org/10.1016/j.geoderma.2022.116123

Wetterskip Fryslân. (2018). *Watersysteemanalyse Opsterlandse Compagnonsvaart*.

Wetterskip Fryslân. (2019). *Watersysteemanalyse Schoterlandse Compagnonsvaart*.
